# Job vacancies at the European Centre for Disease Prevention and Control 

**DOI:** 10.2807/1560-7917.ES.2024.29.27.240704v

**Published:** 2024-07-04

**Authors:** Eurosurveillance editorial team

**Affiliations:** 1 European Centre for Disease Prevention and Control (ECDC), Stockholm, Sweden

The European Centre for Disease Prevention and Control (ECDC) invites applications for the following positions:

- Principal Expert Vaccine-preventable Diseases (2 posts)
- Principal Expert Food- and Water-borne Diseases

The application deadline expires at 12:00 noon on 30 August 2024 (Stockholm time). 

For more information, please visit the ECDC website: https://erecruitment.ecdc.europa.eu/?page=advertisement

